# Collective efficacy in soccer teams: a systematic review

**DOI:** 10.1186/s41155-021-00183-y

**Published:** 2021-06-26

**Authors:** Mylena Aparecida Rodrigues Alves, Marcus Vinicius de Souza Lencina, Mayara Juliana Paes, Joice Mara Facco Stefanello

**Affiliations:** Federal University of Paraná (UFPR), Paraná, Brazil

**Keywords:** Collective efficacy, Group dynamics, Psychometric instrument, Measurement

## Abstract

Collective efficacy, defined as a group’s shared belief about its conjoint capability to organize and execute courses of action, plays a pivotal role in understanding the dynamics of sports teams, since it influences what individuals choose to do as team members, how much they invest in motivational terms to perform actions, how much they work collectively, and for how long they persist despite failure. Through a systematic review, it was investigated how collective efficacy has been assessed in the context of soccer and which indicators, attributes, and psychometric properties have been contemplated in the instruments used. Following the PRISMA guidelines, 22 articles were retrieved through electronic databases (APA PsycINFO; SPORTDiscus; Science Direct; BVS; Web of Science; Scopus; PubMed; and Scielo), using as descriptors, in English, Spanish, and Portuguese, collective efficacy and soccer, combined by the Boolean operators AND and OR. The study did not delimit the initial year of publication for the searches carried out, including all articles found until January 14, 2021 (date of the last update). The following eligibility criteria were adopted: scientific articles published in journals; original studies, which specified the instrument used to assess collective efficacy and carried out with soccer athletes. Five instruments (FCEQ, CEQS, CEI, CEC, and CEQsoccer) that evaluated technical-tactical and psychological attributes associated with collective efficacy in soccer players were identified. In most studies, psychometric properties were restricted to content validity and reliability (internal consistency), and there were no suitable validation processes for the instruments used to measure collective efficacy, which can be considered a limiting factor for understanding this psychological construct in soccer modality.

## Introduction

When we talk about emotions and behaviours in the sports environment, understand the group dynamics and the relationships between group and its members has been highlighted by researchers (Dominski et al., 2018; Fiorese et al., 2019; Trevelin & Alves, 2019; Vilarino, Andrade, Felden, Fomes, & Andrade, 2017; Yeemin, Dias, & Fonseca, 2016) has been highlighted. In collective sports, for example, athletes depend on each other to perform their actions in training and competitions, being this interdependence a valuable condition for the team’s success (Feltz, Short, & Sullivan, 2008; Leo et al., 2019, b).

The Social Cognitive Theory explains this phenomenon based on the concept of human agency (Bandura, 2006; Bandura, Azzi, & Polydoro, 2008). Accordingly, human thinking and behavior are products of the dynamic interrelation between personal, behavioral, and environmental influences. Therefore, the judgment of how capable the team is to perform a task determines the results that this team hopes to achieve from the joint actions (Bandura, 1997).

The shared belief of a group regarding its collective ability to organize and execute courses of action required to produce certain levels of achievement, known as collective efficacy (Bandura, 1997), plays a pivotal role in understanding the dynamics of sports teams (Jowett, Shanmugam, & Caccoulis, 2012), as it influences what individuals choose to accomplish as members of a team (Bandura, 1997; Short, Sullivan, & Feltz, 2005), how much they invest in motivational terms for performing actions, how much they work collectively, and how long they persist despite failure (Bandura, 1997). It is understood, therefore, that team members can share a judgment about their collective competence when they are able to allocate, coordinate, and integrate their resources as a group for a specifically situational demand, producing a successful response (Zaccaro, Blair, Peterson, & Zazanis, 1995).

Different conceptual frameworks (Bandura, 1997; Carron & Eys, 2012; Fuster-Parra, García-Mas, Ponseti, & Leo, 2015) and scientific studies (Bray, 2004; Damato, Grove, Eklund, & Cresswell, 2008; Garza, Ponzanelli, López, Pérez Llantada, & Garcia-Mas, 2015; Leo, Sánchez-Miguel, Sánchez-Oliva, Amado, & García-Calvo, 2011) have highlighted that collective efficacy is one of the most important variables related to performance and success in sports. In the literature, there is the Collective Efficacy Questionnaire for Sports (CEQS) (Short et al., 2005), which evaluates the athletes’ perception about the collective efficacy of their team, and their items were constructed to consider attributes of the psychological order of sports teams, aiming not to specify specific actions of the modalities in the technical aspect (Short et al., 2005). The development of CEQS is consistent with the guidelines of Bandura (2006) for the construction of scales of efficacy.

However, although the great relevance of collective effectiveness is identified in different studies (Bray, 2004; Damato et al., 2008; Garza et al., 2015; Leo et al., 2011), in football, the object of analysis of this systematic review, it is not yet clear how this psychological construct is evaluated. It is important to consider that measures of collective effectiveness aim to meet the characteristics of a specific population, with regard to their functioning, and must be developed for the specific domain of the research area (Bandura, 2006). Thus, it becomes of great relevance to investigate how collective efficacy has been assessed in the sports context, considering the instruments used for assessment, the attributes (physical, technical, tactical, or psychological) contemplated in these measurement instruments, and their psychometric properties (Damato et al., 2008; Garza et al., 2015; Leo et al., 2011; Short et al., 2005). It is worth mentioning that the purpose of these instruments should be the operationalization of constructs or latent traits in behaviors that represent them, thus enabling their empirical observation and scientific analysis (Cronbach, 1996; Pasquali, 2010).

Thus, the present systematic had as specific objectives to investigate how collective efficacy has been assessed in the context of soccer and to analyze which psychometric properties and which indicators and attributes have been considered in the instruments used to analyze this psychological construct.

## Method

This study, classified as a systematic review, used statistics as a tool to identify the psychometric properties of the instruments to be investigated (Pasquali, 2010). Systematic reviews can examine trends in psychological factors related to the performance of athletes and assist in the development of appropriate psychological skills training programs (Krane & Williams, 2006). This review was designed according to the guidelines of the Preferred Reporting Items for Systematic Reviews and Meta-Analyses (PRISMA) (Page et al., 2021). The electronic databases consulted were defined according to the thematic areas that encompass them, as follows: APA (PsychINFO) in the area of psychology; SPORTDiscus in the area of Physical Education; and Science Direct, VHL (Virtual Health Library), Web of Science, Scopus, PubMed, and Scielo (Scientific Electronic Library Online), in the area of Health Sciences.

### Eligibility criteria

The present study included publications in scientific journals and original studies, which specified the instrument used to assess collective efficacy and conducted exclusively with soccer athletes. Course completion papers, dissertations, theses, books, book chapters, conference abstracts, reviews, instrument validation out of the sports context, and studies that did not involve the assessment of collective efficacy in soccer were excluded.

The electronic search strategies were performed using the descriptors collective efficacy (*eficácia coletiva*) and soccer (*futebol*), combined by the Boolean operators AND and OR, with terms enclosed by quotation marks (“”) for compound words, in English, Spanish, and Portuguese (“collective efficacy” OR “eficácia coletiva” OR “eficiencia colectiva”) AND (“soccer” OR “futebol” OR “fútbol”). The searches were started in the first half of 2019 and updated in January 2021, and the initial year of publication was not defined.

### Data extraction and quality assessment

Important characteristics of each study (authors, year of publication, aim, participants, assessment instruments, dimensionality, type of response scale, psychometric properties, indicators, and attributes of collective efficacy) were extracted and recorded in Microsoft Excel. Data are depicted in Tables 1 and 2.

**Table 1:** Characterization of the studies included in the systematic review

Study NR	Authors (year)	Aims	Participants/age group/level	Main results
1	Damato et al. (2008)	To analyze the effect of the absence of an important and unimportant player due to a hypothetical injury on the collective efficacy of a team.	194 male soccer players/16 to 33 years/semi-professional	Following the injury scenario, perseverance collective efficacy perceptions only, significantly decreased following the loss of either player.
2	Price and Weiss (2011)	To examine the relation between leadership behaviors perceived by athletes/team, cohesion, and collective efficacy.	191 female soccer players/14 to 18 years/under-15 and under-18	Canonical correlation analyses revealed that (a) peer leaders were characterized by higher perceived soccer competence, peer acceptance, behavioral conduct, and intrinsic motivation and (b) effective peer leadership was associated with players who reported greater task and social cohesion and collective efficacy.
3	Leo et al. (2001b)	To examine the relationships between the motivational climate created by coaches and peers regarding collective efficacy.	377 male soccer players/average age 24.51 ± 3.73/professional	The mastery climate created by peers and coaches had a significant and positive relationship to collective efficacy.
4	Leo, Sánchez-Miguel, Sánchez-Oliva, Alonso, and García-Calvo (2012)	To examine the evolution of the perception of cohesion, self-efficacy, and collective efficacy among male soccer players over the season and their relation with success expectations.	265 male soccer players/15 to 19 years 15 coaches/29 to 45 years/under-18	The most noteworthy results show that players whose expectations do not match the team’s final performance will experience a negative evolution of their levels of perceived cohesion and efficacy, whereas players whose expectations at the start of the season match the team’s final performance in the classification will maintain their degree of perceived cohesion and efficacy
5	Leo, Sánchez-Miguel, Sánchez-Oliva, Amado, and García-Calvo (2013)	To determine the cohesion and collective efficacy profiles of different male soccer players and measure their differences in terms of success expectations, playing time, and performance.	235 male soccer players/15 to 19 years 15 coaches/29 to 45 years/under-18	Soccer players with higher cohesion and collective efficacy levels belonged to teams that completed the season at the top-level classification. In contrast, athletes with low cohesion and collective efficacy usually played in unsuccessful teams.
6	González-Ponce, Sanchez-Oliva, Amado, and Pulido (2013)	To analyze the relationships between cohesion, collective efficacy, and performance of female soccer players.	66 female soccer players/15 to 33 years/professional	The importance of unity in solving tasks and above all the confidence of the players in the capabilities of the group, as this seems to work in favor of obtaining higher performance by the team.
7	González-Ponce, Sanchez-Oliva, Amado, and Leo (2013)	To explore differences in the motivational climate of teammates and coaches, cohesion, and collective efficacy of players of both sexes.	75 male soccer players e 69 female soccer players/15 to 36 years/professional	Female teams had greater scores in social cohesion than male teams, whereas male teams perceived higher peer performance climate than female teams. Furthermore, either both male and female teams, collective efficacy was related to cohesion and peers and coaches mastery climate.
8	Hampson and Jowett (2014)	To examine the effects of coach leadership and coach-athlete relationship with team efficacy.	112 male soccer players and 38 female soccer players/average age 20.07 ± 1.5/semi-professional and professional	Multiple regression analyses revealed that perceptions of both coach leadership and the coach–athlete relationship predicted variance in team efficacy.
9	Leo, Sánchez-Miguel, Sánchez-Oliva, Amado, and García-Calvo (2014)	To apply a theoretical model evaluating collective efficacy, motivational climate, group cohesion, and their main consequence in performance.	203 male soccer players/18 to 37 years/semi-professional	To optimize perception of collective efficacy and so, increase performance, it seems important that coaches promote strategies to enhance task-related motivational climate and group cohesion in players.
10	Leo, González-Ponce, and Miguel (2015)	To examine the conflict of roles and the conflict between teams as facilitators or debilitators of collective efficacy.	225 female soccer players/15 to 36 years/professional	Group conflicts might have more relevance than role conflict in decreasing confidence in the team’s ability to deal with competition.
11	Leo, González-Ponce, Sánchez-Miguel, Ivarsson, and García-Calvo (2015)	To examine how perceptions of role ambiguity, role conflict, team conflict, and cohesion can predict collective efficacy in sports teams.	320 male soccer players and 210 female soccer players/15 to 39 years/professional	Multilevel modeling analysis showed that perceptions of team conflict and cohesion, at the interpersonal and interteam levels, can predict changes in collective efficacy.
12	Fuster-Parra et al. (2015)	To analyze the team performance and collective efficacy through a Bayesian network.	377 male soccer players/18 to 39 years/semi-professional	The Bayesian network is used to make inferences regarding our problem, and therefore, we obtain some conclusions; among them are as follows: maximizing the team’s performance causes a decrease in collective efficacy and when team’s performance achieves the minimum value it causes an increase in moderate/high values of collective efficacy.
13	Filho, Tenenbaum, and Yang (2015)	To explore the interrelation between cohesion, team mental models (1), collective efficacy (2), and perceived performance potential (PPP).	162 male soccer players and 178 female soccer players/20 to 38 years/professional	The cohesion was found to be an exogenous variable predicting both team mental models and collective efficacy beliefs. Team mental models and collective efficacy were correlated and predicted PPP, which in turn accounted for 59% of the variance of objective performance scores as measured by teams’ season record.
14	Fransen et al*.* (2015)	To analyze the reciprocal relation between team confidence (confidence in results and collective efficacy) and perceived team performance.	Study 1: 134 male soccer players/average age 15.09 ± 0.8/under-17 Study 2: 125 male soccer players/average age 17.3±3.6/under-21	A relationship was found between the perceived performance of the team and the subsequent confidence of the players’ team.
15	Garza et al. (2015)	To assess collective efficacy and teamwork disposition (individual/collective).	112 male soccer players/13 to 27 years/amateur and semi-professional	The players showed more sense of individualism than collectivism.
16	Leo, González-Ponce, Amado, González, and Calvo (2016)	To examine how perceptions of ambiguity and role conflict can predict group cohesion and influence transactional memory and collective efficacy in teams of female soccer players.	225 female soccer players/15 to 36 years/professional	The results suggest that the group leaders in female sports teams will have to make an effort to define the roles of each member of the team to improve the union and group work, because these factors are linked to the capacity of sharing knowledge among group members and the confidence in abilities when facing teamwork.
17	Leo, González-Ponce, Sánchez-Oliva, Amado, and García-Calvo (2016)	To determine the direction of the relationship between cohesion and collective efficacy and its effect on team performance.	146 male soccer players/15 to 18 years/under-18	During pre-season and at the start of the season, team sport coaches should focus on social and task aspects, both individually and at a group level. This would improve the perception of collective team efficacy and lead to better team performance.
18	Atkinson, Short, and Martin (2018)	To examine the relationships between athletes’ perceptions on the effectiveness of their coaches and the collective efficacy of the team.	271 male soccer players/18 to 26 years/semi-professional	A canonical correlation analysis between the variants formed by the Coaching Efficacy Scale subscales and the Collective Efficacy Questionnaire for Sport subscales was statistically significant
19	Leo, González-Ponce, et al. (2019)	To explore the interrelationship between cohesion, transactive memory systems (TMS) and collective efficacy through a conceptual model of cohesion.	557 soccer professionals/16 to 37 years/professional	Task cohesion had a stronger impact on TMS and collective efficacy than social cohesion.
20	Leo, García-Calvo, et al. (2019)	Analyze the number of task, social, and external leaders within sports teams and examine the effectiveness of different leadership structures in men’s and women’s teams.	317 male soccer players/average age 25.25 ± 4.7 214 female soccer players/average age 22.22 ± 4.41/professional	Male teams showed more benefits when having more task and external leaders, while female teams experienced more benefits when having more task and social leaders on the team.
21	Bruton, Shearer, and Mellalieu (2019)	To compare the effects of individual-level observational learning versus team-level observational learning interventions on self-efficacy and collective efficacy beliefs in team sport athletes.	11 male soccer players/average age 21.73 ± 1.51 11 female soccer players/average age 21.94 ± 1.76/amateur, semi-professional and professional	The novel findings of this investigation show that individual-level observational learning, team-level observational learning, and multi-level observational learning interventions can enhance efficacy beliefs in practical contexts and warrant application in groups across domains.
22	Hong and Jeong (2020)	To examine the connection between transformational and authentic leadership of head coaches and team performance, and the mediating role of collective efficacy in this relationship in the context of the Korean Men’s K League.	106 male soccer players/19 to 40 years/professional	The transformational and authentic leadership of head coaches both had a positive effect on players’ collective efficacy, which has a positive effect on team performance.
23	Mertens et al. (2021)	To explore how leadership structures among athletes within sports teams evolve over the course of a season.	460 male soccer players/average age 23.5 ± 4.55/semi-professional	Findings suggest that leadership structures in sports teams can change considerably over the course of the competitive season, thereby challenging the classic view of stable, vertical leadership structures.

*Study NR*, numeric reference of study

**Table 2:** Characterization of the instruments used in the studies included in this systematic review

NR study	Evaluation instrument	Instrument dimensionality and type of response scale	Psychometric properties of instruments	Collective efficacy indicators	Attributes of collective efficacy
3	Football Collective Efficacy Questionnaire (FCEQ)	One-dimensional Likert-type response scale (1–5)	Content validity CFA Internal consistency	Reliability in specific collective tasks in attack, defense, and offensive and defensive transitions	Technical-tactical and psychological
4					
5					
6					
7					
9					
10					
11					
12					
16					
17					
19					
20					
2	Collective Efficacy Questionnaire for Sports (CEQS)	Multidimensional Likert-type response scale (0–9)	Content validity EFA CFA Convergent/discriminant validity Criterion validity Internal consistency	Ability, preparation, effort, persistence, and unity	Physical, technical-tactical and psychological
8					
13					
14					
18					
21					
23					
1	Collective Efficacy Inventory (CEI)	Two-dimensional Likert-type response scale (0–10)	Content validity Internal consistency	Perseverance collective efficacy, skills collective efficacy, and offensive and defensive transitions	Physical, technical-tactical and psychological
15	Cuestionario de Eficácia Colectiva Percibida (CEC) FÚTBOL	Multidimensional Likert-type response scale (1–5)	Content validity EFA Internal consistency	Efficacy of counterattack, defense, finalizing actions/transition, offensive, and concentration	Technical-tactical and psychological
22	Collective Efficacy Questionnaire for Soccer (CEQsoccer)	Multidimensional Likert-type response scale (1–5)	Content validity EFA CFA Convergent validity Internal consistency	Team strength, sufficient training, leader confidence, and effective communication.	Physical, technical-tactical and psychological

*EFA*, exploratory factor analysis; *CFA*, confirmatory factor analysis; *NR study*, numerical reference of the study

The quality analysis of the selected studies was based on the studies of Pasquali (2009), Carretero-Dios and Pérez (2005), and Primi, Muniz, and Nunes (2009) that establish quality indicators for the construction and validation of psychometric instruments, being content validity, construct validity (exploratory factor analysis, confirmatory factor analysis, and convergent/discriminant validity), criterion validity (concurrent or predictive), and reliability (internal consistency and temporal stability). To control the risk of bias, the selection and evaluation of all studies were conducted by two authors independently, being the differences compared and resolved with the participation of a third author for reaching a consensus (Page et al., 2021).

## Results

The initial search yielded a total of 219 studies. Of these, 16 studies were excluded: dissertations (n = 5), books (n = 4), book chapters (n = 5), and conference abstracts (n = 2), resulting in 196 studies. By reading the titles of the articles, duplicate publications were also excluded (n = 79), thus retaining 117 studies. Posteriorly, researches that had no relation to the sports context (n = 42) were excluded, totaling 75 manuscripts.

**Fig. 1:**
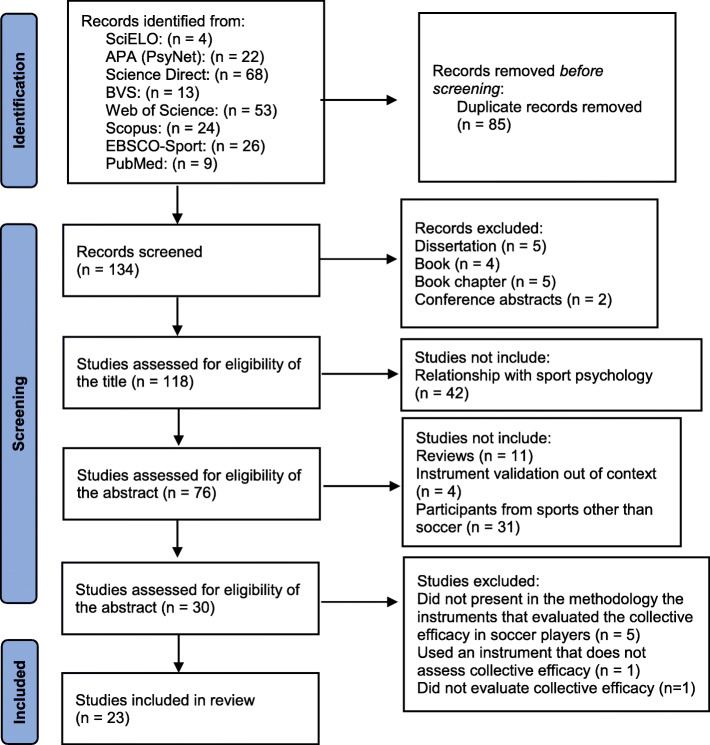
PRISMA diagram describing the selection process of the reviewed articles

After reading the abstracts, 46 articles were excluded because they were review papers (n = 11) and instrument validation out of the sports context (n = 4) and that did not involve the assessment of collective efficacy in soccer (n = 31), hence resulting in 29 studies for full-text assessment. In this step, five studies were excluded for not mentioning the instruments that evaluated collective efficacy in soccer players and one study that used an instrument that did not assess collective efficacy. The selection process of the reviewed articles is illustrated in Fig. 1.

Table 1 summarizes the characterization of the 23 articles selected for this systematic review.

The largest number of publications was found on collective efficacy in soccer concentrated in 2015 (26.1%), while the others were published in 2008 (4.3%), 2011 (8.7%), 2012 (4.3%), 2013 (13%), 2014 (8.7%), 2016 (8.7%), 2018 (4.3%), 2019 (13%), 2020 (4.3%), and 2021 (4.3%).

Most studies (91.3%) analyzed the relationship of collective efficacy with other variables, being group cohesion and sports performance the most investigated (65.2%). The participants had, on average, 260.3 ± 151.1 soccer players (minimum age of 13 years old and maximum of 40 years old). Six studies (26.1%) included athletes of both sexes, four (17.4%) only female athletes, and 13 studies (56.5%) only male athletes.

### Main results

Table 2 reveals the instruments used to measure the collective efficacy in soccer athletes, the dimensionality, type of response scale, psychometric properties, indicators, and the collective efficacy attributes evaluated in these instruments.

The 23 studies used standardized instruments, with Likert-type response scales: the Football Collective Efficacy Questionnaire (FCEQ), Collective Efficacy Questionnaire for Sports (CEQS), Collective Efficacy Inventory (CEI), Cuestionario de Eficácia Colectiva Percibida (CEC) – FÚTBOL, and Collective Efficacy Questionnaire for Soccer (CEQsoccer). CEQS (Short et al., 2005), CEC (Garza et al., 2015), and CEQsoccer (Yoo & Lim, 2009) are characterized as multidimensional measures, with five (ability, preparation, effort, persistence, and unity), four (counterattack, defense, attack, and finalizing actions/transitions), and four (team strength, sufficient training, leader confidence, and effective communication) dimensions, respectively. CEI (Damato et al., 2008) is a two-dimensional instrument (perseverance collective efficacy and skills collective efficacy), and FCEQ (Leo et al., 2011) is one-dimensional (collective tasks between attack and defense).

To assess the psychometric properties, the recommended criteria were adopted for the construction and validation of psychometric instruments (Carretero-Dios & Pérez, 2005; Pasquali, 2009; Primi et al., 2009), establishing as quality indicators the presence of (a) content validity, (b) construct validity (exploratory factor analysis, confirmatory factor analysis, and convergent/discriminant validity), (c) criterion validity (concurrent or predictive), and (d) reliability (internal consistency and temporal stability).

CEQS was the instrument that contemplated a higher number of psychometric properties (six of the seven established as quality criteria): content validity, exploratory factor analysis, confirmatory factor analysis, convergent/discriminant validity, internal consistency, and concurrent validity. Contemplating five psychometric properties is the CEQsoccer (content validity, exploratory factor analysis, confirmatory factor analysis, convergent validity, and internal consistency). FCEQ (content validity, confirmatory factor analysis, and internal consistency) and CEC (content validity, exploratory factor analysis, and internal consistency) considered three psychometric properties. In turn, CEI evaluated only two psychometric properties (content validity and internal consistency). Thus, content validity and internal consistency were the most contemplated psychometric properties in the instruments used in the studies that assessed collective efficacy in soccer (100% of the research), followed by factor analysis (95.6% of the research).

The indicators and attributes of collective efficacy determined in the context of soccer included (a) physical attributes (indicators of strength, speed, and endurance), evaluated in 39.1% of the studies; (b) technical attributes (indicators related to the fundamentals of the sport), analyzed in 100% of the studies; (c) tactical attributes (indicators related to specific collective tasks of attack/defense, counterattack, offensive, and defensive transitions), determined in 100% of the studies; and (d) psychological attributes (indicators related to confidence, effective communication, concentration, perseverance, preparation, effort, persistence, and union), evaluated in 100% of the studies. Specific indicators for assessing collective efficacy in soccer (counterattacks, offensive, and defensive transitions) were found in four instruments (FCEQ, CEQsoccer, CEC, and CEI), covered in 69.6% of the studies.

## Discussion

The main objective of this work was to investigate how collective efficacy has been assessed in the context of soccer and which psychometric properties, indicators, and attributes were considered in the instruments used.

Instruments elaborated and validated specifically for the soccer context (FCEQ, CEI, CEC, and CEQsoccer) or created to evaluate this construct in different collective sports modalities (CEQS) have been used in the reviewed studies. According to Fransen, Mertens, Feltz, and Boen (2017), the best way to measure a group’s beliefs in relation to collective skills is to ask team members about their perceptions of the group’s skills rather than their individual skills.

The importance of using properly validated instruments for the assessment of psychological constructs is defended by several scientific researchers, and they should be developed with the purpose of organizing and explaining aspects of the construct in question and also to obtain reliable results, thereby reducing the chances of errors and bias (Pais-Ribeiro, 2013; Pasquali, 2010; Souza, Alexandre, & Guirardello, 2017).

In the present systematic review, indicators of content validity, construct validity, and reliability were the most contemplated. The content validity (Pasquali, 2009) was verified through the qualitative analysis of the items among specialists with knowledge about the construct assessed in the five instruments (FCEQ, CEI, CEC, CEQS, and CEQsoccer). The construct validity was measured in CEQS (used in seven studies) and FCEQ (used in 12 studies) instruments, being tested, mainly, through confirmatory factor analysis, considered the most important psychometric analysis in the construction of an assessment instrument, allowing to represent, through statistical calculations, an observational behavior of a latent trait (Pasquali, 2009). The reliability of the instruments used to determine collective efficacy was verified through internal consistency in order to demonstrate the accuracy of the tests, using Cronbach’s alpha coefficient to estimate the correlation of each test item and the rest of the items, as well as the correlation between the total score of the items (Pasquali, 2009).

All instruments utilized a Likert-type scale to assess collective efficacy, with a predominance of the 5-point scale (Fuster-Parra et al., 2015; Garza et al., 2015; González-Ponce, Sanchez-Oliva, Amado, & Leo, 2013; González-Ponce, Sanchez-Oliva, Amado, & Pulido, 2013; Hong & Jeong, 2020; Leo et al., 2012; Leo et al., 2013; Leo et al., 2014; Leo, González-Ponce, et al., 2019; Leo, González-Ponce, Amado, et al., 2016; Leo, González-Ponce, & Miguel, 2015; Leo, González-Ponce, Sánchez-Miguel, et al., 2015; Leo, González-Ponce, Sánchez-Oliva, et al., 2016), which allows the respondent to feel more comfortable in expressing his opinion (Coelho & Esteves, 2007), for having a neutral response option.

Specific instruments for assessing collective efficacy in soccer players were used in 14 studies (Damato et al., 2008; Fuster-Parra et al., 2015; Garza et al., 2015; González-Ponce, Sanchez-Oliva, Amado, & Leo, 2013; González-Ponce, Sanchez-Oliva, Amado, & Pulido, 2013; Hong & Jeong, 2020; Leo et al., 2012; Leo et al., 2013; Leo et al., 2014; Leo, González-Ponce, et al., 2019; Leo, González-Ponce, Amado, et al., 2016; Leo, González-Ponce, & Miguel, 2015; Leo, González-Ponce, Sánchez-Miguel, et al., 2015; Leo, González-Ponce, Sánchez-Oliva, et al., 2016). Two studies (Damato et al., 2008; Garza et al., 2015) used instruments developed exclusively to achieve the objective of the study. These instruments aimed to provide a comprehensive evaluation of the construct and different variables of soccer development, namely physical, technical-tactical, and psychological aspects (González-Víllora, Serra-Olivares, Pastor-Vicedo, & da Costa, 2015).

The studies prioritized the analysis of technical attributes (fundamentals of the modality), tactical (specific collective tasks of attack/defense, counterattack, offensive, and defensive transitions), and psychological (confidence, effective communication, concentration, perseverance, preparation, effort, persistence, and union). The physical aspects were the least contemplated in the instruments used, which may be a limiting factor in the studies recorded herein. According to Bandura (1997), beliefs of collective efficacy must reflect the group’s capabilities as substantial implications for its effort and performance in tasks that demand interaction to achieve success, thus requiring a wide range of performances.

## Conclusions

In summary, the results of the present study indicate the use of four instruments developed and validated specifically to assess collective efficacy in the context of soccer, measuring physical, technical, tactical, and psychological attributes. The most mentioned psychometric properties of the studies were restricted to indicators of content validity and reliability (internal consistency). No important analyses were found, such as predictive validity, test-retest analysis, and longitudinal analyses in the reviewed studies, which points to incomplete and sometimes inadequate validation processes with regard to the instruments used to measure collective efficacy. The low number of instruments specifically validated for the context of soccer, besides the restriction of the measured psychometric properties (content validity and reliability), may be considered limiting factors for understanding this psychological construct, because it can imply the reliable evaluation of the beliefs of collective efficacy in this modality.

Remarkably, the restriction of searches to the context of soccer, the delimitation of only three languages (English, Spanish, and Portuguese), and the non-inclusion of additional studies beyond the selected works from the databases represent the major limitations of this systematic review. Thus, it is suggested to consider these aspects in future investigations.

Finally, as practical implications, it is worthwhile noting that the present review raises relevant questions regarding the tools used to assess this psychological construct, hence suggesting that future research should focus on expanding the assessment of the psychometric properties of instruments developed specifically for the sport, also contemplating the use of more robust analyses. Such actions may contribute to the reduction of biases in the evaluation of the conduct of athletes and assist coaches in the development and improvement of skills related to the group dynamics of sports teams, which are markedly important to achieve the expected success.

## Data Availability

All data generated or analyzed during this study are included in this published article.
